# The presence of the proteolysis-inducing factor in urine does not predict the malignancy of a pancreatic tumour

**DOI:** 10.1186/1471-230X-5-20

**Published:** 2005-06-21

**Authors:** Niels Teich, Jörg Kleeff, Herbert Lochs, Joachim Mössner, Volker Keim, Helmut Friess, Johann Ockenga

**Affiliations:** 1 Universität Leipzig, Medizinische Klinik und Poliklinik II, Leipzig, Germany; 2 Universität Heidelberg, Chirurgische Universitätsklinik, Heidelberg, Germany; 3 Charité Berlin, Medizinische Klinik und Poliklinik, Gastroenterologie, Hepatologie und Endokrinologie, Berlin, Germany

## Abstract

**Background:**

The proteolysis-inducing factor (PIF) was identified as a tumour product in various gastrointestinal cancers. A previous study in pancreatic cancer patients suggested PIF expression as a tumour marker, which is not related to tumour size. We hypothesized that PIF could be a useful marker to exclude benign pancreatic tumors, as chronic pancreatitis with a pancreatic mass.

**Methods:**

Urine of patients with a pancreatic mass of uncertain malignancy was investigated for PIF expression by Western blot. Sufficient urine protein for analysis was available in 59 patients. The diagnosis was established by histology in 54 patients and by follow up in five patients with chronic pancreatitis. In addition, serum CA19-9 was measured.

**Results:**

The sensitivity (specifity) for the detection of a malignant pancreatic tumour was 90% (75%) and 54% (71%) for CA19-9 and PIF, respectively. The sensitivity (specifity) for the distinction of pancreatic cancer from chronic pancreatitis was 89% (80%) and 57% (63%) for CA19-9 and PIF, respectively.

**Conclusion:**

Evaluation of PIF in urine is of no diagnostic value in patients with a pancreatic mass of unknown malignancy.

## Background

Distinction between benign and malignant pancreatic tumours is still difficult, despite significant progress in imaging techniques. Patients with chronic pancreatitis are at increased risk to develop pancreatic cancer [1]. Pancreatic inflammation, as observed in chronic pancreatitis, can be mistaken on imaging as cancer and inversely. Serum carbohydrate antigen 19-9 (CA19-9) levels are elevated in 80% of pancreatic cancer patients, but can also be increased in 20% of patients with chronic pancreatitis [2]. An accurate and non-invasive test to differentiate pancreatic cancer from chronic pancreatitis is not available.

Cancer frequently induces cachexia, but not all cancer patients will develop cachexia. This is not necessarily a late phenomenon in tumour progression. It may be present at diagnosis and may be the leading symptom that induces search for a tumour. A study of pancreatic cancer patients revealed that patients had lost a median of about 14% of their usual body weight at the time of diagnosis, and that this weight loss was progressive, increasing to a median of 25% at the time of the last assessment [3]. The central role in this process of – predominant – skeletal muscle waste seems to play the proteolysis-inducing factor (PIF) [4].

PIF is a 24-kDa sulphated glycoprotein synthesized by cachexia-inducing murine and human tumours, which induces catabolism of myofibrillar proteins in skeletal muscle via a direct stimulation of the proteasome pathway in muscle cells [5]. Administration of PIF to normal mice leads to a rapid decrease in body weight, which is based primarily on a loss of skeletal muscle mass [4,6].

PIF is expressed in a variety of gastrointestinal cancers [7]. It was detected in the urine of 44 from 55 pancreatic cancer patients, who had a significantly greater total weight loss and rate of weight loss than patients whose urine did not contain PIF. Interestingly, PIF expression was not dependent on cancer stage, but seemed to be a qualitative marker of pancreatic cancer: even stage 2 tumours expressed PIF in 83 per cent [8].

PIF has not been investigated in benign pancreatic diseases. We therefore hypothesized that PIF production in patients with a pancreatic tumour would clearly indicate the malignant nature of the disease. Special attention was paid to a clear distinction to a pancreatic mass caused by chronic pancreatitis.

## Methods

100 patients with a pancreatic mass of uncertain malignancy (detected by computed tomography, magnet resonance tomography, ultrasound and/or endoscopic retrograde cholangiopancreaticography) were investigated. Patients with UICC stage 4 (locally extended and metastasised disease) were not enrolled. After collection of urine from all study participants, we investigated 30 ml urine for the presence of PIF with a specific mouse monoclonal antibody in a Western blot setting as described recently [4,8]. In brief, urine protein was precipitated with ammonium sulphate and dialysed against water between 12 and 15 hours (overnight) with a molecular weight cut-off of 10 kD (Slide-A-Lyzer Dialysis Cassette). 5 μg of concentrated samples were separated with sodium dodecylsulphate polyacrylamide gel electrophoresis and subsequently blotted on nitrocellulose membranes. After incubation with 10 μg/ml of the mouse monoclonal PIF antibody (provided with courtesy by MJ Tisdale) and streptavidine horseradish peroxidase conjugate, the bands were detected using the Fluorescent ECL Plus system (Amersham). In addition, CA19-9 in serum was measured with an electrochemiluminescence immunoassay (CA19-9^®^Roche Diagnostics or ADVIA Centaur^®^, Bayer Healthcare), the upper limit of normal was 22 U/ml. The ethics committees of the three participating centres approved this protocol.

## Results and discussion

**Figure 1:**
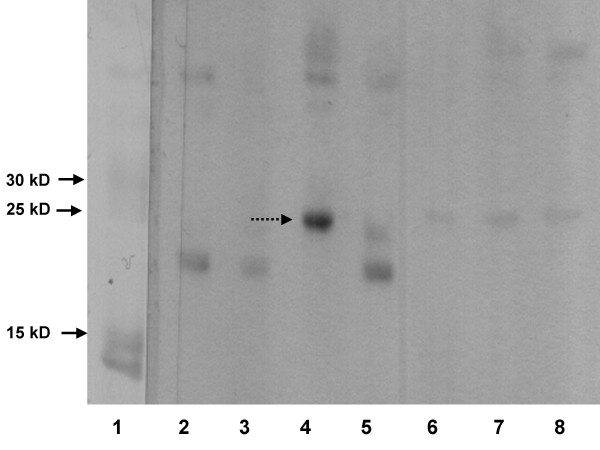
Western blot analysis for PIF after urinary protein extraction. Lane 1: Rainbow^® ^recombinant protein molecular weight marker (#RPN 800, Amersham Life Science); lane 2: healthy control person, lanes 3 and 5: chronic pancreatitis, PIF not detected, lanes 4 and 7: pancreatic cancer, UICC stage 1 and 2, respectively, PIF detected (24 kD, broken line); lanes 6: chronic pancreatitis, PIF detected; lane 8: positive control (pancreatic cancer, UICC stage 4)

**Figure 2:**
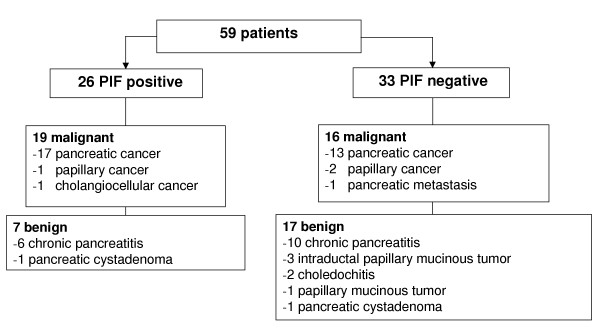
Diagnoses in PIF positive and PIF negative patients

The analysis failed in 41 patients due to insufficient amount of urinary protein after dialysis. In 59 patients, PIF Western blot was successfully performed (figure 1). This cohort was subsequently assessed: it included 31 male and 28 female patients (median age 59 years, range 33–89 years). The diagnoses of the patients were shown in figure 2. Final diagnosis was done by histology in 54 patients or follow up for at least one year in five patients with chronic pancreatitis, who did not undergo surgery.

The diagnostic values of PIF for the detection of a malignant pancreatic tumour and with special attention to pancreatic cancer are shown in table 1. Summarizing all 59 patients, the median CA19-9 values in patients with benign or malignant pancreatic tumours were 16.5 (standard deviation (SD): 141) and 477 (SD: 22173 U/l), respectively (p < 0.05). The median CA19-9 values in patients with chronic pancreatitis and pancreatic cancer were 15.0 (SD: 18) and 478.5 U/l (SD: 22022), respectively (p < 0.05). The analysis of CA19-9 in dependence on the presence of PIF in urine in PIF negative patients revealed 22.5 U/l (SD: 742) and 580.5 U/l in PIF positive patients (SD: 25203) (p < 0.05). PIF was detected in two patients with CA19-9 negative pancreatic cancer, but was not detectable in 16 pancreatic cancer patients (15 of them with elevated CA19-9).

This is the first attempt to evaluate the proteolysis-inducing factor as a diagnostic marker of pancreatic cancer in patients with a potentially resectable pancreatic tumour. We found a weak association with malignancy, but the diagnostic value to distinguish benign from malignant pancreatic tumours is lower than CA19-9. PIF seems to be of a rather limited importance to answer this question in every-day care.

A major drawback of our data is the high amount of patients with insufficient urinary protein after extraction and dialysis. For practical reasons in this multi-centre collaboration, we tried to investigate PIF in a small urine sample, as initially suggested by Todorov et al. [4]. Our problem could be potentially solved by the collection of higher urine volumes in further studies.

PIF was detected in 6 patients with chronic pancreatitis without evidence for a malignancy by 12 months follow up or histology. This raises the question about the origin of PIF in these patients. Although the majority of investigations found PIF expression only in tumour tissue, the expression of PIF in non-malignant tissue has been also reported [7]. The primary role of PIF seems to be in regulation of development [9]. After cloning of the cDNA for PIF, a human homologue – the human cachexia associated protein (HCAP) – has been identified, which is minimally expressed in normal tissues [10,11]. It can be speculated, that the ongoing inflammation in patients with chronic pancreatitis is capable to generate PIF. This could explain the low specifity of PIF to detect pancreatic malignancies in our study. Three patients with pancreatic cancer had normal CA19-9 values, two of them were PIF-positive. A sequential testing of PIF in the case of a CA19-9-negative pancreatic tumour may be beneficial but has to be considered cautiously based on these small patient numbers. However, even this approach would fail to detect all pancreatic cancer patients in our study.

Although our data indicate that PIF is not helpful as a diagnostic marker of pancreatic cancer, a benefit may be the early identification of patients who need nutritional intervention [12]. In previous studies the detection of PIF was associated with prior weight loss. PIF induces an increase of the ubiquitin – proteasome activity resulting in protein catabolism. Administration of the polyunsaturated fatty acid eicosapentaenoic acid (EPA) attenuates protein degradation by antagonizing the PIF induced up regulation of the ubiquitin – proteasome proteolytic pathway in cachectic tumour bearing mice [13]. A first randomised placebo – controlled trial in patients with pancreatic cancer suggest that a EPA enriched oral supplement has the potential to induce a net gain of weight, lean body mass and improvement of quality of life [14].

## Conclusion

PIF is not superior to the established tumour marker CA19-9 to distinguish benign from malignant pancreatic tumours. Further investigations should clarify whether the onset of PIF expression in the long-term follow-up of chronic pancreatitis patients is associated with early malignancy and whether it precedes morphologic and clinical signs of pancreatic cancer. In future, the evaluation of PIF as an indicator for early nutritional intervention seems to be warranted.

## Abbreviations

proteolysis-inducing factor PIF

pancreatic cancer PaCa

chronic pancreatitis CP

carbohydrate antigen 19-9 CA19-9

eicosapentaenoic acid EPA

standard deviation SD

## Competing interests

The author(s) declare that they have no competing interests.

## Authors' contributions

N.T and J.O. had the initial idea and initiated the study. N.T. co-ordinated the laboratory analysis. All authors ascertained patients in this multicenter investigation, wrote and discussed the final manuscript.

## Pre-publication history

The pre-publication history for this paper can be accessed here:



## Figures and Tables

**Table 1:** Diagnostic values of PIF in comparison to CA19-9 to discriminate malignant vs. benign and pancreatic cancer (PaCa) vs. chronic pancreatitis (CP).

	**all malignant vs. all benign**		**PaCa vs. CP**	
	**PIF**	**CA19-9**	**PIF**	**CA19-9**

**Sensitivity**	54%	90%	57%	89%
**Specifity**	71%	75%	63%	80%
**positive predictive value**	73%	88%	74%	93%
**negative predictive value**	52%	80%	44%	73%
